# Pre-Historic and Recent Vicariance Events Shape Genetic Structure and Diversity in Endangered Lion-Tailed Macaque in the Western Ghats: Implications for Conservation

**DOI:** 10.1371/journal.pone.0142597

**Published:** 2015-11-11

**Authors:** Muthuvarmadam S. Ram, Minal Marne, Ajay Gaur, Honnavalli N. Kumara, Mewa Singh, Ajith Kumar, Govindhaswamy Umapathy

**Affiliations:** 1 Laboratory for the Conservation of Endangered Species, CSIR-Centre for Cellular and Molecular Biology, Uppal Road, Hyderabad, 500007, India; 2 Salim Ali Centre for Ornithology and Natural History, Coimbatore, 641108, India; 3 Biopsychology Laboratory, and Institution of Excellence, University of Mysore, Mysore, 570006, India; 4 Wildlife Conservation Society-India, Centre for Wildlife Studies, Bangalore, 560070, India; Chinese Academy of Forestry, CHINA

## Abstract

Genetic isolation of populations is a potent force that helps shape the course of evolution. However, small populations in isolation, especially in fragmented landscapes, are known to lose genetic variability, suffer from inbreeding depression and become genetically differentiated among themselves. In this study, we assessed the genetic diversity of lion-tailed macaques (*Macaca silenus*) inhabiting the fragmented landscape of Anamalai hills and examined the genetic structure of the species across its distributional range in the Western Ghats. We sequenced around 900 bases of DNA covering two mitochondrial regions–hypervariable region-I and partial mitochondrial cytochrome b–from individuals sampled both from wild and captivity, constructed and dated phylogenetic trees. We found that the lion-tailed macaque troops in the isolated forest patches in Anamalai hills have depleted mitochondrial DNA diversity compared to troops in larger and continuous forests. Our results also revealed an ancient divergence in the lion-tailed macaque into two distinct populations across the Palghat gap, dating to 2.11 million years ago. In light of our findings, we make a few suggestions on the management of wild and captive populations.

## Introduction

The contemporary spatial distribution of genetic diversity in a species is a function of its life history traits on the one hand and pre-historic and recent vicariance events on the other. Social and dispersal systems have major influences on spatial distribution of genetic diversity [1]. Across contiguous habitat, species with female-biased dispersal system, such as the northern muriqui (*Brachyteles hypoxanthus*) [2] and the black-and-gold howler (*Alouatta caraya*) [3], show little structuring of mitochondrial DNA (mtDNA) unlike in female-philopatric macaques [4] which have structured spatial distribution of mtDNA.

The pre-historic vicariance events that influence spatial distribution of genetic diversity include paleo-climatic events, such as glaciations [5], and geological events acting as barriers [6]. These events produce genetic divergence among groups in contiguous forests in species with female-biased and male-biased dispersal [3]. The altitudinal difference in haplotype diversity in the Japanese macaque (*Macaca fuscata*) in Yakushima Island [7], genetic differences between rhesus macaques (*Macaca mulatta*) in India and China and among populations within China [8], and population level differences in mtDNA between the Moroccan and Algerian populations of the Barbary macaque (*Macaca sylvanus*) [9] probably occurred due to volcanic activity, dispersal barriers and climatic events respectively.

Among the major recent vicariance events threatening biodiversity is human-caused habitat fragmentation [10–12]. The decrease in population and the fragmentation and isolation of remaining population can lead to loss of genetic diversity and increased differentiation at short distances. Both of these have been studied in several taxa using mtDNA [13–14] which better reflects rapid changes in genetic diversity. Studies on primates show that dispersal systems mediate genetic impacts of habitat fragmentation. In the same fragmented habitat, the Tana River red colobus (*Procolobus rufomitratus*) with male and female dispersal retained more mtDNA haplotypes and had greater nucleotide diversity than the Tana River mangabey (*Cercocebus galeritus*) with only male dispersal [15]. Both the species, however, showed differentiation between forest patches, which increased with geographical distance only in the case of the mangabey.

While arboreal animals and less mobile terrestrial animals seem to be invariably affected, the genetic impacts of habitat fragmentation on highly mobile animals seem to be more variable [16–17]. Genetic diversity in a frugivorous bat, *Dermanura watsoni*, was greater in the larger forest fragments and in fragments with suitable habitats in the surrounding matrix [18]. In mammalian carnivores in fragmented landscapes, the genetic diversity of source populations from which immigrants arrive is more important than fragment size or population size [19]. In the absence of mobility across the matrix between forest fragments, populations of carnivores in forest fragments, invariably small due to their low densities, can rapidly lose genetic diversity, e.g. Atlantic Forest jaguars (*Panthera onca*) [20] and Florida black bear (*Ursus americanus floridanus*) [21].

Thus, contemporary spatial distribution of genetic diversity is highly variable depending upon so many factors that it might be species and locality specific. In this paper, we examine the spatial distribution of mtDNA in the lion-tailed macaque (*Macaca silenus*) at two temporal levels. We examine the extent of genetic differentiation between two populations on either side of an ecological barrier- the Palghat gap- that predates the arrival of the lion-tailed macaque in the Western Ghats mountain ranges in peninsular India to which it is endemic. Parts of the Western Ghats have undergone extensive habitat loss and fragmentation, starting from 1860’s. Therefore, we also examine the nature and extent of loss of genetic diversity as indicated by mtDNA.

### The lion-tailed macaque

The lion-tailed macaque is an obligate rainforest primate endemic to the Western Ghats mountain range. Compared to other macaques, the lion-tailed macaque has a late age at first birth (6 years), long inter-birth interval (30 months), but high survival (80% to adulthood). Population growth rate is, therefore, low. Mean group size in contiguous forests is about 18 individuals and most of these are single-male groups [22]. However, in isolated small fragments, groups may become as large as about 100 animals, with more than 10 adult males and 30 adult females [23].

The Western Ghats mountain range extends for about 1600 km from the southern tip of India to the state of Gujarat, running almost parallel to the west coast. The most prominent break in the mountain range is the Palghat gap which is about 40 km wide. This gap is about 500 million years old [24] and thus predates the arrival of mammals and birds in the Western Ghats. Although the loss of forest connectivity across this gap was recent and due to human occupation, genetic differentiation between populations north and south of the gap has been reported not only in taxa which have low mobility (e.g. white-bellied short-wing) [25], but also in Asian elephants [26]. Therefore, we expected to see genetic differentiation between populations south and north of the Palghat gap.

The loss and fragmentation of forests in the Western Ghats predates that in most other parts of the tropics, and has its roots in the establishment of tea plantations starting from 1860’s. Even between 1920 and 1990, there was a 40% loss of forest cover and a fourfold increase in the number of forest fragments in the Western Ghats [27]. The rainforest was among the most severely fragmented habitats; Anamalai hills was one such range. The Valparai plateau in the Anamalai hills now has nearly 220 km^2^ of tea plantations with more than 20 rainforest patches and the plateau is surrounded by protected areas with contiguous rainforests. The total population of the lion-tailed macaque in Anamalai hills is about 800 animals and nearly half of them are in forest fragments in the tea plantations [23]. These forest fragments were formed in early 1900’s [28]. This landscape, therefore, is an ideal system to examine the impacts of habitat fragmentation on genetic differentiation. Previous studies on lion-tailed macaques in this landscape have examined population dynamics [23], demography [29,30], feeding behavior [31] and parasites [32].

In this study, we used mtDNA sequences (i) to examine genetic diversity and structure of the lion-tailed macaque across its distribution in the Western Ghats and (ii) to assess the genetic diversity of the population in the fragmented forests of Anamalai hills with reference to a continuous population in the adjacent Nelliyampathy hills. Estimation of genetic diversity in fragmented landscapes will help us employ better management practices and policies in both in-situ and ex-situ conservation efforts [33]. Besides, an understanding of the overall genetic structure may provide us with valuable insights into the evolutionary history of the species and the presence of barriers to gene flow.

## Methods

### Study area

**Fig 1:**
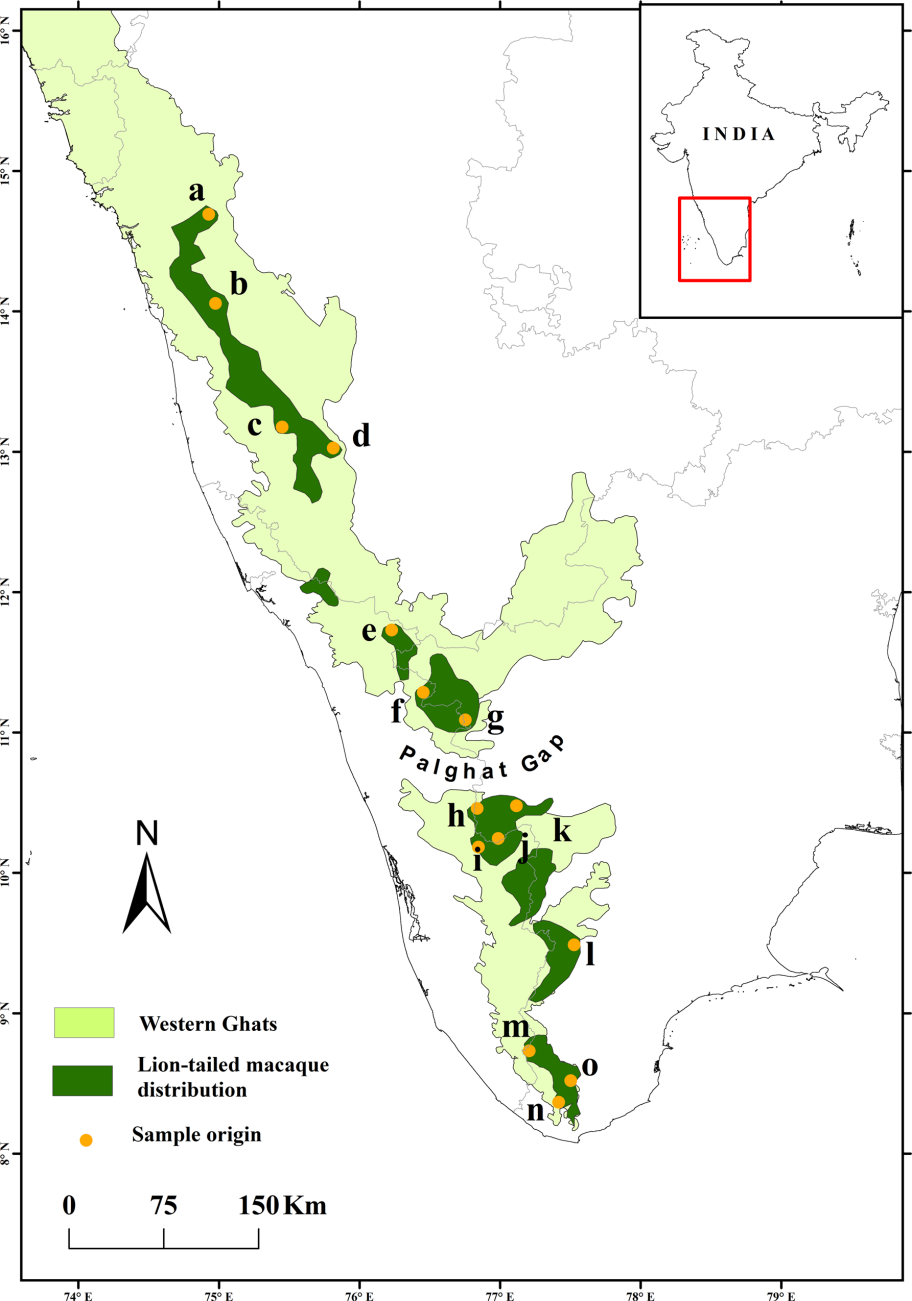
The disjoint distribution of lion-tailed macaques in the Western Ghats. The wild origins of samples used in this study are marked: a- Aghanashini, b- Kundapur, c- Karkala, d- Kudremukh, e- Muthanga, f- Vazhikkadavu, g- Silent Valley, h- Nelliyampathy, i Kodanad, j- Vazhachal, k- Anamalai, l- Meghamalai, m- Kollam, n- Amboori and o- Kalakkad.

Lion-tailed macaques occur in the wet evergreen forests from the central to the southernmost regions of the Western Ghats. To examine the genetic structure of the lion-tailed macaque population across its distribution in the Western Ghats, fecal samples of individuals were collected from Aghanashini, Karkala, Kundapur and Kudremukh in Karnataka state, Muthanga, Vazhikkadavu, Silent Valley, Nelliyampathy, Kodanad, Vazhachal, Kollam and Amboori in Kerala State, and Anamalai, Meghamalai and Kalakkad in Tamil Nadu State (Fig 1).

**Fig 2:**
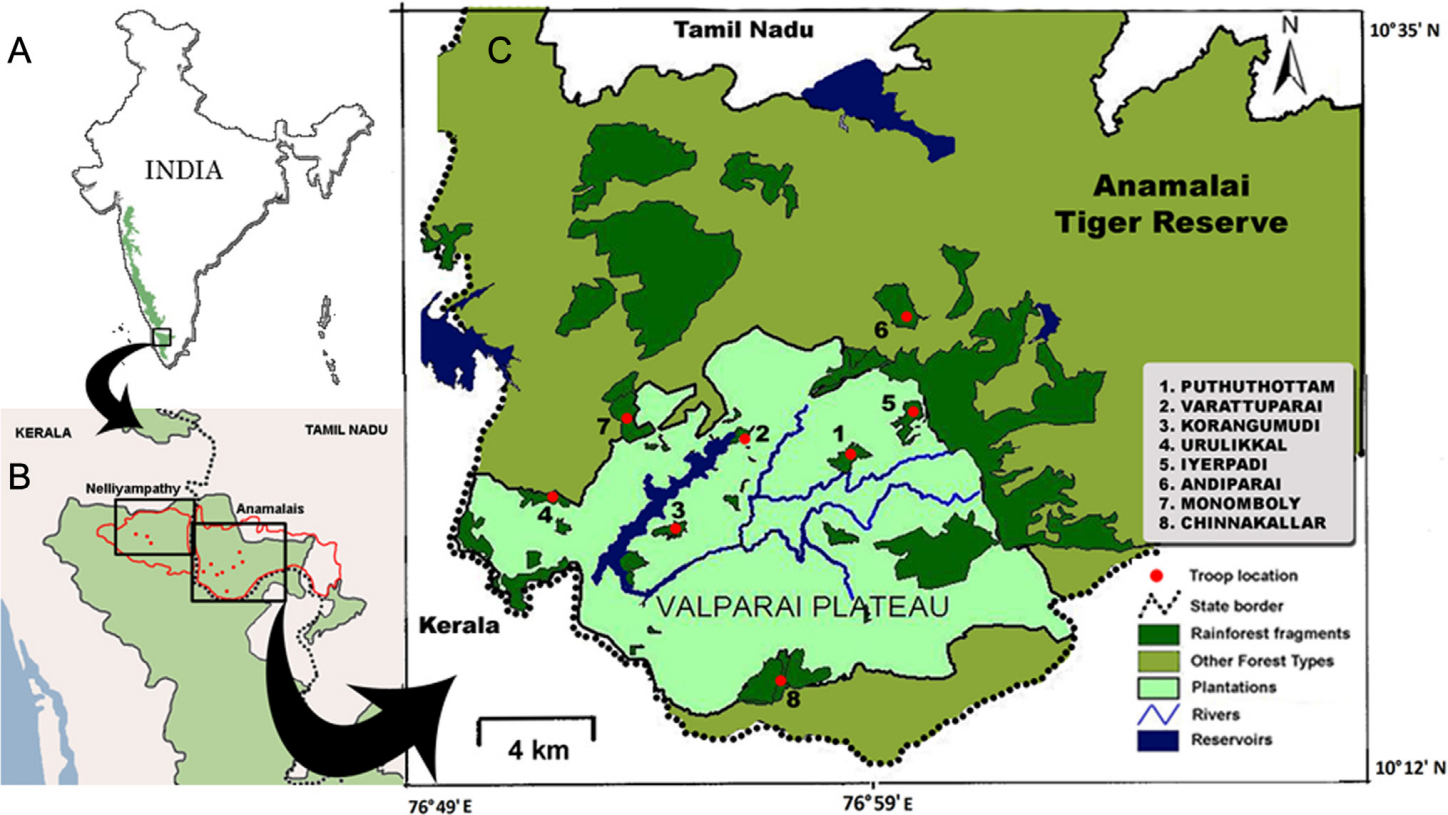
Map showing study areas. Sampling locations of Anamalai and Nelliyampathy in the Western Ghats (A); Three sampled groups in Nelliyampathy (B) and a detailed map showing sampled groups from eight isolated rainforest fragments in Anamalai hills (C).

To understand the impact of habitat fragmentation on the genetic diversity of the lion-tailed macaque populations, a study was undertaken in the rainforest fragments of Anamalai Tiger Reserve, Anamalai hills, Western Ghats, India (Fig 2). Eight forest fragments, which vary in time since isolation, were chosen for the study. Four fragments (Andiparai, Korangumudi, Varattuparai and Urulikkal) contain a single troop and the rest (Puthuthottam, Chinnakallar, Monomboly and Iyerpadi) contain at least two troops each of lion-tailed macaques. For comparison, we also collected samples from the reserve forest of Nelliyampathy where more than 10 groups are found as a single, continuous population (Fig 2).

### Sample collection

All sample collections were done during the years 2010–2012. Eighty-eight fecal samples were collected from 8 forest fragments in Anamalai hills (Fig 2) and 15 fecal samples from 3 troops in Nelliyampathy hills. In addition, samples were collected from Kudremukh-Someshwara (n = 4), Sirsi-Honnavara/Aghanashini (n = 14), Silent Valley National Park (n = 4), Vazhachal (n = 1), Vazhikkadavu (n = 3) and Meghamalai (n = 1). Kudremukh-Someshwara samples were from two troops, whereas the rest were from single troops. Collection was carried out without direct interaction with the macaques and causing no disturbance to the habitat. Permissions to collect lion-tailed macaque fecal samples from Tamil Nadu, Kerala and Karnataka states were granted by the Principal Chief Conservator of Forests, Tamil Nadu Forest Department, Chennai 600 015 (Ref. No. WL 5/58890/2008, dated 2^nd^ September, 2009), the Principal Chief Conservator of Forests (Wildlife), Kerala Forest Department, Thiruvananthapuram 695 014 (Ref. No. WL12-9371/10, dated 3^rd^ January, 2010) and the Principal Chief Conservator of Forests, Karnataka Forest Department, Bengaluru 560 003 (Ref. No. B3/WL/CR-8/2008-2009) respectively. Fresh feces were collected by following the troops and the feces were stored in 15 mL or 50 mL falcon tubes containing 90% ethanol at room temperature until DNA isolation.

Twenty-three blood samples from captive lion-tailed macaques were also collected in EDTA vacutainers by qualified veterinarians of Mysore, Thiruvananthapuram and Chennai Zoos by physically restraining the macaques (Permission Letter from Central Zoo Authority of India, Ministry of Environment, Forests & Climate Change, Government of India, vide Ref. No. 9-2/2005-CZA(M) Vol III, dated 3^rd^ January, 2011). Information on the origin of these macaques was collected from studbook records. These animals, part of the Conservation Breeding Programme initiated and coordinated by the Central Zoo Authority of India (CZA), were housed as per CZA norms and fed twice a day. Two to three mL of blood was collected and transported at 4°C to the lab and stored until DNA isolation. All experiments were performed in accordance with the guidelines of the Central Zoo Authority of India, Ministry of Environment, Forests & Climate Change Government of India and the study was also approved by the Institutional Animal Ethics Committee of the Centre for Cellular and Molecular Biology, Hyderabad, India.

### DNA isolation, amplification and sequencing

The ethanol was drained out of the sample collection tubes and the fecal samples were placed on a petriplate for drying at room temperature. After treating the sample with 1X PBS for 4 hours, the resultant mixture was centrifuged and the pellet was lysed by incubating overnight at 50°C in 1.5 mL lysis buffer (50 mM Tris HCl, 10 mM EDTA and 150 mM NaCl) containing 2% sodium dodecyl sulfate and 200 μg proteinase K. Genomic DNA was extracted from the lysate using standard phenol-chloroform method [34], i.e., the lysate was treated twice with an equal-volume mixture of phenol and chloroform and once with only chloroform before being precipitated using iso-propanol. The DNA was dissolved in double distilled water and then purified using GeneClean Kit (MPbio) following the supplier’s recommendations and stored at 4°C in the elution buffer provided. For EDTA blood samples, 100 μL of blood was lysed and DNA was isolated using standard phenol-chloroform method [34] and stored at 4°C in TE buffer.

Macaque-specific primers, D1 (5’ GTACACTGGCCTTGTAAACC 3’) and D3 (5’ CTTATTTAAGGGGAACGTGTGG 3’), for hypervariable region-I (HVR-I) region were designed from conserved domains of reference sequences of *M*. *fascicularis*, *M*. *mulatta* and *M*. *sylvanus* available in GenBank (accession numbers: NC_012670, NC_005943 and NC_002764, respectively) using the primer designing tool, Primer3 [35,36]. This primer pair amplifies from position 15901 to 16553 of *M*. *mulatta* (NC_005943) and gives an amplicon of ca. 650 bp in the lion-tailed macaque. Universal primers designed by Verma and Singh [37] were used for amplifying a partial region of mitochondrial cytochrome b (cytb).

PCR reactions were performed in 10 μL reaction volumes of 1X PCR buffer, 2.5 mM of MgCl_2_, 1X BSA, 0.25 μM each of forward and reverse primers and 0.75 units of Taq polymerase (ExTaq HS DNA Polymerase, Takara Bio Inc.). PCRs were performed with initial denaturation at 95°C for 10’, 35 cycles each of denaturation at 95°C for 20”, annealing at 52°C for 20” and extension at 72°C for 30”, followed by a final extension at 72°C for 10’. PCR products were visualized on 2% agarose gel and the DNA extracted with Gel Elution Kit (BioServe India). To prevent cross-sample contamination the workspace was cleaned with bleach and ethanol prior to each extraction. Pre and post-PCR works were carried out in separate, dedicated spaces and all extractions and PCRs were done with a negative control.

Sequencing was repeated thrice with both forward and reverse primers. Where the results were contentious, PCR was repeated to resolve the issue. Sequencing was carried out in ABI 3730XL sequencer using BigDye Terminator cycle sequencing kit (Applied Biosystems). Excluding the primer region, we generated sequences of a maximum of 613 bases spanning tRNA-thr (partial), tRNA-pro and partial HVR-I (519 bases) and sequences of a maximum of 521 bases of mitochondrial cytochrome b (cytb) were also generated. The presence of nuclear mitochondrial inserts (numts) is unlikely in our samples as the fecal samples are richer in mitochondrial DNA and no extremely variant sequences were observed. However, we screened cytb sequences for numts by translating into amino acid sequences to detect stop codons.

### Genetic analysis

The identity of the samples was checked using BLAST and all sequences showing maximum similarity to *M*. *nemestrina* or *M*. *leonina* were deemed to be of lion-tailed macaque origin as no control region sequence of the same was available in the database. Subsequently, after the generation of voucher lion-tailed macaque sequences, the identities were verified by comparing with the voucher. The forward and reverse sequences were aligned and manually edited for base-calling errors using CodonCode Aligner 2.0.6 (http://www.codoncode.com/). A second editing step was performed where all the edited sequences were aligned and the trace sequences were checked for editing-errors at all sites of mismatch. The edited sequences were realigned using CLUSTAL W [38] with default settings as implemented in MEGA5 [39].

For assessing genetic diversity, a dataset containing 103 sequences and 519 bases of HVR-I was used. Insertions and deletions were excluded from the analysis. Genetic variability was estimated in terms of haplotype diversity (h) and nucleotide diversity (π). Tajima’s D was also estimated to infer if the data departs from the ideal, neutrally evolving population. All diversity estimations were done in DnaSP 5.10 [40]

For estimating genetic structure, a combined dataset containing 495 bases of HVR-I and 394 bases of cytb was used. Since some of the HVR-I sequences generated low quality bases towards the end, the sequences had to be truncated to 495 bases from 519 bases to accommodate the maximum number of haplotypes in the analysis. Corresponding sequences of *M*. *sylvanus* (GenBank accession number: KJ567054), *M*. *mulatta* (KJ567051 and JQ821843), *M*. *cyclopis* (KM023192), *M*. *fuscata* (KM401548), *M*. *fascicularis* (KM851010 and KM851007), *M*. *thibetana* (KJ567056 and EU294187) and *M*. *assamensis* (KF990122), cut from complete mitochondrial genome sequences available in GenBank, were used as outgroups. The simplest model of sequence evolution that best explains the pattern of nucleotide variations in the dataset was determined using jModelTest 2.1.3 [41,42]. Based on Bayesian Information Criterion (BIC) and Decision Theory (DT) values, HKY+I+G (Hasegawa-Kishino-Yano substitution model with proportion of invariant sites = 0.391 and gamma shape parameter = 0.741) was chosen as the best fitting model.

Bayesian inference trees were reconstructed using Mr.Bayes 3.1.2 [43]. Two parallel MCMC runs of 5 million generations each were performed, each with three heated chains and one cold chain. The trees were sampled every 250 generations. The convergence of the two independent runs was assessed from the split frequency of standard deviation that was calculated once every 500 generations. The first 25% of generations were discarded as burn-in. The run parameters of the remaining generations were summarized and the uncorrelated potential scale reduction factor (PSRF) values for all parameters were confirmed to be equal to one before proceeding to summarize the trees to give a 50% majority rule consensus tree with posterior probability values for each branch. Maximum Likelihood (ML) trees were reconstructed using raxmlGUI 1.3[44] with the model GTRGAMMAI and 10,000 rapid bootstrap replications.

BEAST 1.7.5 [45] was used to estimate the divergence ages. The same dataset was used but with additional outgroup sequences from GenBank of *Papio ursinus* (accession numbers: JX946204 and JX946205), *P*. *anubis* (JX946197 and JX946198) and *Theropithecus gelada* (KC757412 and FJ785426). To estimate the divergence times, the following fossil-based calibration dates adopted from Liedigk et al. [46] were applied as normal priors to constrain the ages of two nodes: The *Papio-Theropithecus* split at 5.0 Ma with a 95% credibility interval (CI) of 1.5 Ma and the split between African and Asian macaques at 5.5 Ma with a 95% CI of 1.0 Ma. A relaxed uncorrelated lognormal model of lineage variation and Birth-Death process prior for branching rates were assumed. Four independent MCMC runs with 25 million generations each were performed and trees were sampled every 1,000 generations. Adequacy of 10% burn-in and the stability of the runs were checked using TRACER 1.5 [47]. The sampling distributions of the four runs were combined (25% burn-in) using LogCombiner 1.7.5 [45] and the resulting 75,004 trees were summarized to generate a maximum clade credibility tree using TreeAnnotator 1.7.5 [45]. The tree was visualized with the divergence ages at each node and 95% CI values as node bars using FigTree 1.2.2 [48].

## Results

### Genetic structure and diversity in lion-tailed macaque

One-hundred-and-forty-seven fecal samples of lion-tailed macaques were collected from across the Western Ghats (Fig 1). We amplified and sequenced 519 bp of the mtDNA control region (HVR-I). Thirty-one unique haplotypes were detected (GenBank accession numbers: KR265223-KR265253), which were characterized by a total of 128 polymorphic sites (32.65%) of which 108 were parsimony informative. All point mutations were transitions; there were three insertions and three deletions. The mean nucleotide difference for the entire Western Ghats population was 40.23. Three-hundred-and-ninety-five bases of mitochondrial cytochrome B (cytb) were also sequenced and analyzed for all the above samples. Fourteen haplotypes were observed (GenBank accession numbers: KR265209-KR265222) with 25 polymorphic sites (6.35%), for entire Western Ghats population, of which two were transversions and the rest were transitions. Twenty sites were parsimony informative. The unique haplotypes of HVR-I were used to construct a neighbor-joining tree to understand relationship between the haplotypes. Two distinct clades were observed with high branch support; one containing all samples from the north of Palghat gap and the other from its south. The genetic diversity of the south-of-Palghat population was higher (mean pairwise nucleotide difference—29.1 for HVR-I, 6.2 for cytb) than the north (19.8 for HVR-I, 2.8 for cytb) (Table 1). The average genetic distance between populations north and south of Palghat gap (56.0 for HVR-I, 11.9 for cytb) was almost double that of the genetic distance for individuals within these populations.

**Table 1:** Genetic distance within and between north and south clades.

Population	Average no. of nucleotide differences / Mean pairwise nucleotide distance	
	HVR-I	cytb
**Within north-of-Palghat**	19.834 / 0.041	2.762 / 0.007
**Within south-of-Palghat**	29.132 / 0.060	6.190 / 0.016
**Between north and south**	56.004 / 0.115	11.939 / 0.030

**Fig 3:**
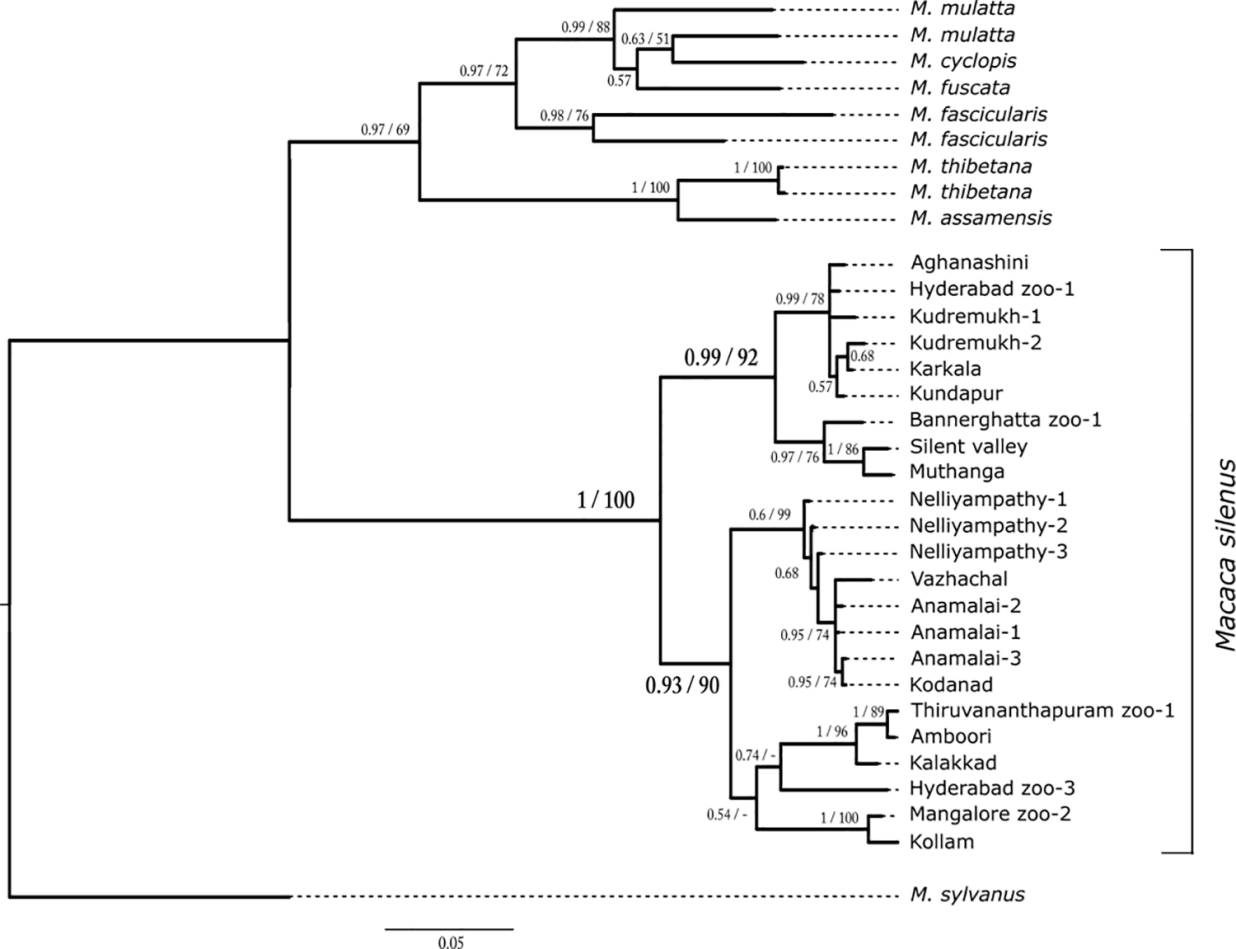
Bayesian inference tree. A Bayesian inference tree was reconstructed from 893 bases (including gaps) of macaque mitochondrial DNA sequences. Leaves are labelled with the samples’ wild origins (see Fig 1) and individuals of unknown wild origin are labelled by their zoos of origin. More than one haplotype from a region is indicated by a trailing number. The numbers at each node are the Bayesian posterior probabilities and bootstrap values of the corresponding nodes in the maximum likelihood tree. Nodes with a hyphen in place of the bootstrap value were not present in the ML tree. Bootstrap values less than 50 are not shown.

Phylogenetic trees were reconstructed using Bayesian inference (Fig 3) and maximum likelihood methods (S1 Fig). Both trees showed a divergence into north- and south-of-Palghat gap clades) with good support.

**Fig 4:**
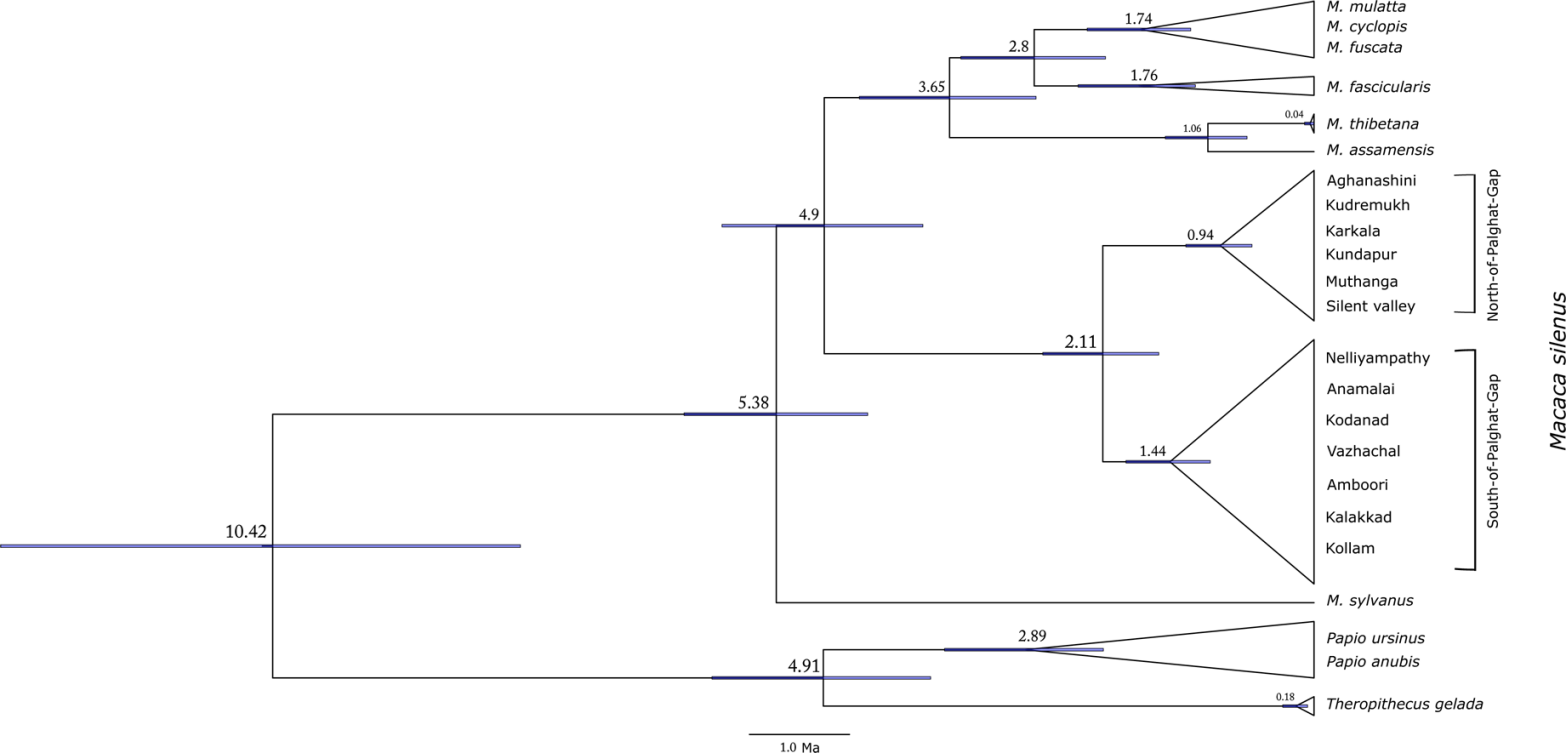
Divergence dating of the north-south split in lion-tailed macaque. Divergence dating tree of the lion-tailed macaque and outgroup taxa was reconstructed from 893 bases of mitochondrial DNA. The values at each node indicate the estimated mean divergence dates in million years from present (Ma). The *Papio*-*Theropithecus* split and the split between *Macaca sylvanus* and the Asian macaques were used as calibration points.

For dating the divergences of these clades, a Bayesian inference tree was constructed and the divergence ages were calibrated using fossil-based dates for the *Papio-Theropithecus* and the African-Asian macaques splits in BEAST 1.7.5 (Fig 4). We observed that the mean time to the most recent common ancestor (TMRCA) of north and south clades was 2.11 Ma (Fig 4). The estimated mean divergence dates and their credibility intervals are given in Table 2. The estimated mean divergence dates for the major splits within the Papionini tribe were within the range previously reported [46].

**Table 2:** Divergence times of the most recent common ancestors calculated from 893 bases of mitochondrial DNA, using two fossil calibrations.

Most Recent Common Ancestor	Estimated Mean Divergence Date (Ma) and 95% confidence intervals	Estimated mean divergence date (Ma) by Liedigk et al. [46]
*Macaca-(Papio+Theropithecus)*	10.415 (7.9368–13.1342)	10.69 (8.24–13.20)
*Papio-Theropithecus*	4.9077(3.8347–6.021)	5.20 (4.04–6.41)
*Macaca sylvanus-*Asian macaques	5.3774(4.4644–6.3005)	5.93 (4.95–6.93)
Fascicularis-Sinica group of macaque	3.645(2.7804–4.5473)	3.97 (3.09–4.90)
*M*. *silenus* north-south	2.112(1.5527–2.7119)	N.A

### Genetic diversity in the fragmented population of Anamalai hills

Eighty-eight samples were collected from eight fragments in Anamalai hills and amplified and sequenced 519 bp of HVR-I. Three haplotypes were obtained, with a single major haplotype (Anm1) represented by 84 sequences from all the eight fragments and two minor haplotypes (Anm2 and Anm3) from three fragments which are in the periphery of the fragmented Anamalai hills landscape (Fig 2). To compare this fragmented population with a large continuous population, 15 samples were collected from three groups (65 individuals) from Nelliyampathy hills (Fig 2) and three haplotypes were observed. Higher haplotype diversity (Hd) and nucleotide diversity (π) were observed in the Nelliyampathy population compared to the Anamalai population (Table 3). Tests of neutrality were not significant for both groups, indicating that these populations have not undergone any recent population expansion or bottleneck.

**Table 3:** Comparison of hypervariable region-I diversity in lion-tailed macaques of Anamalai and Nelliyampathy hills.

	Anamalai hills	Nelliyampathy hills
**No. of fragments**	8	1
**No. of sequences**	88	15
**No. of polymorphic sites**	2	3 (+ 1 insertion/deletion)
**No. of haplotypes**	3	3
**Haplotype diversity (Hd)**	0.0886	0.5333
**Nucleotide diversity (π)**	0.00017	0.00188
**Tajima's D**	-1.22056 (not significant)	0.46509 (not significant)

## Discussion

### Genetic structure and diversity of lion-tailed macaques in the Western Ghats

In the present study, 25.9% of 495 bases of HVR-I region was found to be polymorphic reflecting healthy genetic diversity in the lion-tailed macaque across its range in the Western Ghats.

The lion-tailed macaque in the Western Ghats shows an ancient split across the Palghat gap which dates back to 2.11 Ma. Similar observations of divergence across the Palghat gap have been reported in the Asian elephant [26], the white-bellied shortwing [25], 10 other species of birds endemic to the Western Ghats [49] and the anuran genus *Nyctibatrachus* [50]. The estimated dates of spilt in these taxa vary from 0.23 Ma (Asian elephant) to 40 Ma (*Nyctibatrachus spp*.*)*. Of the 10 species of birds whose divergences across the Palghat gap were dated by Robin et al. [49], 5 have TMRCAs between 2.5 and 1 Ma. The Palghat gap was formed in the Cambrian (around 500 Ma) by tectonic shearing forces, and hence the geological activity associated with its formation is too ancient to affect the distribution of terrestrial animals. More recent Pleistocene climatic perturbations [51–53] have been attributed for the disjunct distribution of species in the Indian subcontinent [54,55] and in the Western Ghats [25,49,50].

Overall genetic diversity of the lion-tailed macaque across its entire distribution is comparable to other macaques. In rhesus macaque, 31% of 544 bases of HVR-I was found to be variable [8].In rhesus macaque, the genetic distance, using HVR-I sequences between Indian and Chinese subspecies was found to range between 0.067 and 0.089 [8]. While in the Japanese macaque, the genetic distance between Yakushima subspecies (*M*. *fuscata yakui*) and the Kyushu haplotypes was 0.047 [7]. In this study, the genetic distance between north and south of the Palghat gap was 0.115 (Table 1), which is higher than the difference among the rhesus and Japanese macaque subspecies. Although opinions currently differ on whether or not mtDNA phylogeny alone can be used to delineate subspecies [56–59], there is more support for early identification of Evolutionarily Significant Units (ESU) in the case of endangered animals, especially until a more detailed picture of the true phylogeny emerges. For example, a study of Variable Number Tandem Repeats in remnant populations of the critically endangered giant panda reported significant genetic differentiation between two populations and it was suggested solely based on molecular data that the two populations be considered as separate subspecies, and that one of the subspecies itself be divided into several management units [60]. Thus, morphological variations alone may not be the criteria for considering ESUs for conserving an endangered species. With wide genetic differentiation between north and south Palghat gap populations, they may be considered as two separate ESUs.

### Effect of fragmentation on genetic diversity of lion-tailed macaques in Anamalai hills

Lion-tailed macaques in the fragmented forests of Anamalai hills have significantly low mtDNA diversity compared to the adjoining large and continuous population of Nelliyampathy hills. Similar reductions in genetic diversity in isolated forest patches as compared to those in continuous forests have been reported in gorillas [61] and giant white-tailed rats [62]. Positive correlation between the fragment size and mtDNA diversity have been found in northern muriqui [2] and toads [13]. In Barbary macaques on Gibraltar, concordant results from both microsatellite and mtDNA analyses have shown that even proximally occurring fragments can impede gene flow, thereby producing unexpected genetic structuring in fragmented populations [63]. Similarly, low genetic diversities have been found in isolated populations of macaques [7,64]. Such reductions in genetic diversity may also occur due to bottleneck events [7], genetic drift or recent population expansion [65]. On the other hand, populations derived from a small founder population have also shown markedly low diversity in long-tailed macaques found in Mauritius [64]. In the Anamalai hills, 8 groups (approximately 260 individuals) in five isolated forest fragments are completely isolated thus no exchange of individuals takes place among them since lion-tailed macaques never disperse through non-canopy matrices [66]. Furthermore, these forest fragments were isolated since 1920s [28] with no gene flow from other contiguous forests resulting in low mtDNA diversity. In addition, all these groups might have also descended originally from a single group resulting in low genetic diversity. In the case of Nelliyampathy, in spite of reduction in forest cover in the recent past [67], genetic diversity is still higher than the isolated, fragmented populations because these groups (more than 10) live in a large contiguous forest resulting in greater gene flow among them.

Of the three haplotypes found in the Anamalai hills, one haplotype (Anm1) was found in all forest fragments whereas the other two (Anm2 and Anm3) were confined only to the peripheral forest fragments (Monomboly, Andiparai and Chinnakallar), (Fig 2). One of the possible explanations for this may be that Anm2 and Anm3 have formed by movement of individuals from adjacent fragments using non-rainforest canopy, whereas lack of any canopy connectivity to the five isolated fragments (Puthuthottam, Korangumudi, Varattuparai, Iyerpadi and Urulikkal) has prevented genetic exchange with other groups resulting in a single haplotype. The other reason could be the extinction of the minor haplotypes (Anm2 and Anm3) in these five isolated forest fragments due to genetic drift [68]. The present study demonstrates that anthropogenic habitat modification affects lion-tailed macaque movement causing reduction in its genetic diversity. However, this study used mitochondrial markers that examine only maternally inherited genetic variation, and therefore role of male dispersal is not accounted for. Further studies should be carried out using nuclear markers to investigate male mediated gene flow among fragmented and isolated populations.

### Conservation implications

Lion-tailed macaques of the Anamalai hills, which form almost 20% of the entire wild population, have very low genetic diversity due to habitat fragmentation. Furthermore, lion-tailed macaques in isolated forest fragments have not been observed using non-canopy matrices around them [69]. This prevents gene flow between them and with large and contiguous populations thus leading to low genetic diversity and accumulation of deleterious mutations, which might pose a severe threat to their long-term survival in these fragmented landscapes. This observation is in accordance with high infant mortality and low survivability among young ones in the small and isolated fragments [29]. Furthermore, in a recent study it was observed that these isolated populations have a high prevalence of gastrointestinal parasites [32], which along with low genetic diversity would negatively affect survival of these isolated population in the long run. Therefore, it is recommended that some of these isolated fragments should be connected by planting food trees that would establish canopy corridors between them to facilitate gene flow.

The present study clearly demonstrates the presence of two genetically distinct populations of lion-tailed macaques across the Palghat gap. These populations can be considered as separate conservation units. Furthermore, we suggest that they should be treated separately in captive breeding programs and any reintroductions to the wild should be done after ascertaining the correct geographic origin of the individuals.

## Supporting Information

S1 Fig Maximum Likelihood tree. Maximum Likelihood tree reconstructed from 893 bases (including gaps) of macaque mitochondrial DNA sequences using raxmlGUI 1.3. Leaves are labelled with the samples’ wild origins (see Fig 1) and individuals of unknown wild origin are labelled by their zoos of origin. More than one haplotype from a region is indicated by a trailing number. The number at each node is the bootstrap support for that node. Bootstrap values less than 50 are not shown.
(TIF)
