# Supplementary figures and images for: Pre-Historic and Recent Vicariance Events Shape Genetic Structure and Diversity in Endangered Lion-Tailed Macaque in the Western Ghats: Implications for Conservation
Source: PLoS One. 2015 Nov 11;10(11):e0142597. doi: 10.1371/journal.pone.0142597 (PMC4641736; doi:10.1371/journal.pone.0142597)

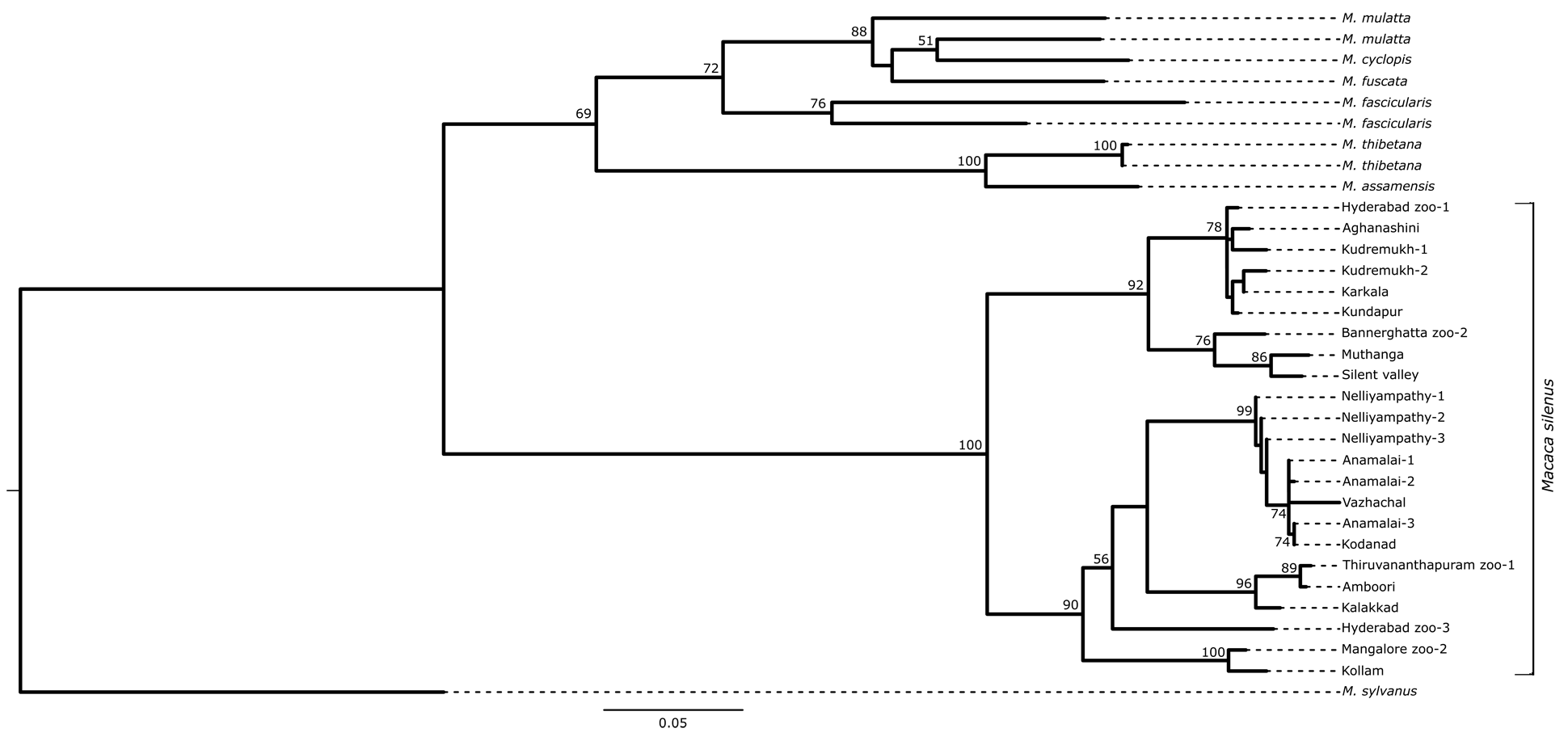

Supplement: S1 Fig — Maximum Likelihood tree reconstructed from 893 bases (including gaps) of macaque mitochondrial DNA sequences using raxmlGUI 1.3. Leaves are labelled with the samples’ wild origins (see Fig 1) and individuals of unknown wild origin are labelled by their zoos of origin. More than one haplotype from a region is indicated by a trailing number. The number at each node is the bootstrap support for that node. Bootstrap values less than 50 are not shown. (TIF) [file pone.0142597.s001.tif]
